# New Inventions

**Published:** 1912-10

**Authors:** 


### NEW INVENTIONS

---

W. J. Robb and W. H. Welch, Bristol, Eng. Assignors to Banner Motore, Ltd., Bristol, Eng. 1,033,939. Internal Combustion Engine.

Henry Sieben, Kansas City, Mo. Assignor to Fire and Gas Appliance Co. 1,033,958 Gas Controlling Apparatus.

S. S. Wyer, Columbus, Ohio. Assignor to The Jeffrey Mfg. Co 1,034,053. Gasolene Rock Drill.

G. R. Blair, Topeka, Kansas. Assignor to S A. Kinne and J. N. Catreu, Topeka, Knsas. 1,034,056. Oil Burner.

W. J. Bergens, Cleveland, Ohio. Assignor to C. L. Terry and J. A. Rommhardt, Cleveland, Ohio. 1,034,204. Thermostatic Compound Valve.

G. H. Tomlinson, Chicago, Ill. Assignor to Standard Alcohol Co., New York, N. Y. 1,032,440. Process of Producing Fermentable Sugars from Lignocellulose.

E. P. Thieringer, New York, N. Y. 1,032,518. Nipple Shield.

E. A. Kern, West New York, N .J. 1,032,610. Poison-Indicating Bottle.

Lorenz Ach, Mannheim-Waldhof, Ger. Assignor to C. F. Boehringer & Soehne, Mannheim-Waldhof, Ger. 1,032,642. Tasteless Quinin Compound and Process of Making Same.

C. F. Snyder, Camillus, N. Y. 1,032,712. Rectal or Pile Supporter.

Otto Schonherr and Johannes Hessberger, Fiskaa, Norway. Assignors to Badische Anilin and Soda Fabrik, Ludwigshafen-on-the-Rhine, Ger. 1,032,782. Process and Apparatus for the Production of Compounds of Nitrogen.

J. T. Keough, Los Angeles, Cal. 1,032,840. Vibratory Dilator.

Julius Bueb, Dessau, Ger. 1,032,988. Process for Obtaining Cyanogen and its Compounds.

J. C. Poore, Chicago, Ill. 1,037,309. Nursing Bottle.

Louis Witt, San Leandro, Cal. 1,037,392. Hospital Bed Appliance.

H. J. Collis, Taunton, Mass. 1,037,441. Ankle Support and Protector.

Rudolf Tambach, Ludwigshafen-on-the-Rhine, Ger. Assignor to The Firm of Knoll & Co. ,Ludwigshafen-on-the-Rhine, Ger. 1,037,685. Case in Derivatives and Process for the Manufacture of the Same.

O. E. Eklund, F. G. Folberth, and W. M. Folberth, San Francisco, Cal. 1,035,954. Inhaler.

Paul Flemming, Hamburg, Germany. 1,036,087. Halogenphenol alkali Salts and Process for the Manufacture of Same.

Herman Haberland and Walter Schaefer, Zacherndorf, Germany. Assignors to The Firm of Salzbergwerk Neu-Stassfurt und Theilnehmer, Zacherndorf, Ger. 1,036,224. Process of Making Penta and Hexachlorethanes.

C. J. Kintner, New York, N. Y. 1,036,262. Syringe adapted to use liquids under pressure.

Jno. T. Davis, Alameda, Calif. 1,023,244. Distillation Apparatus.

Sam'l Morris Lillie, Philadelphia, Pa. 1,023,257. Starch-Conversion Process.

Ralph Scott Swinton, Linden, N. J. 1,023,281. Manufacture of Volatile Organic Acids from their Calcium Salts.

Wm. T. Henderson, Laurinburg, N. C. 1,023,646. Sterilizer.

Miecislaw Marcks, Lubeck, Germany. 1,023,213. Dental Impression Cup.

Perry S. Bauer, Chicago, Ills. 1,023,236. Adhesive Plaster Spool.

Chancy A. Frees, New York, N. Y. 1,023,247. Artificial Leg.

Herbert A. Pullen, Buffalo, N. Y. 1,023,273. Dental Regulator.

Lillian M. Bender, Seattle, Wash. 1,023,358. Face Mask.

Reinhard Roesch, San Jose, California. 1,023,396. Invalid Bed.

Peter C. McEwen and Fred'k W. Young, Detroit, Michigan. 1,023,435. Hernial Truss Pad.

Robert Wahl, Chicago, Ills. 1,023,448. Manufacture of Fermented Malt Beverages.

Michael E. O'Reilly, Portland, Oregon. 1,023,478. Suspensory.

Ralcy Husted Bell, New York, N. Y. 1,023,499. Collapsible Capsule.

Jean Marie Perigny, Lyon, France. 1,023,528. Manufacture of Cream of Tartar.

Geneva Morgan, Thomaston, Me. 1,023,672. Nursing Bottle Attachment.

Gretel J. Pass, Grenada, Miss. 1,023,677. Toilet Hood.

Jas. Walsh and Robt. Dawson Kay, London, England. 1,023,-693. Infant Pacifier.

Ernest E. Adams and Jno. L. Dudek, Schuyler, Nebr. 1,023,-705. Pig Forceps.

Arthur Aubriot Pons, New York, N. Y. 1,023,756. Exercising Apparatus.

W. D. Baker, Indianapolis, Ind. 1,036,540. Adjustable Atomizer.

J. A. Coultrup, Colorado Springs, Colo. 1,036,573. Adjusting-Table for Chiropractors.

W. B. Eastburn, Washington, D. C. 1,036,588. Poison-Indictor.

Ernest Hoennicke, Dresden, Germany. 1,036,622. Process for Producing Thyroid-Gland Extracts.

Heinrich Horlein, Vorwinkel, Near Elberfeld, Germany. Assignor to Farbenfabriken vorm. Friedr. Bayer & Co., Elberfeld, Ger. 1,036,624. Phenylethylmalonic.

Heinrich Horlein, Vorwinkel, Near Elberfeld, Ger. Aissignors to Farbenfabriken vorm. Friedr. Bayer & Co., Elberfeld, Ger. 1,036,625. Phenylethylcyanoacetic Ester.

P. J. McElroy, Cambridge, Mass. Assignor to The Randall-Faichney Co., Boston, Mass. 1,036,672. Piston for Hypodermic Syringes, etc.

Emil Von Portheim, Prague, Austria-Hungary. 1,036,705. Method for the Production of Anhydrous Hudrosulfites.

Wm. Rintoul and A. G. Innes, Stevenson, Scotland. Assignor to E. I. du Pont de Nemours Powder Co., Wilmington, Del. 1,036,715. Process for Condensing Glycerin.

John Sabo, Cleveland, Ohio. 1,036,785. Depilator.

G. W. White, San Antonio, Texas. 1,036,759. Inhaler.

Ludwig Benda, Frankfort of the Main, Ger. Assignor to Farbwerke vorm. Meister Lucius and Burning, Hochst on the Main, Ger. 1,036,784. 5 Nitro 2 Aminobenzene 1 Arsinic Acid.

Chas. Blagburn, Antioch, Cal. 1,036,788. Process of Obtaining Substantially Pure Nitrogen from the Air.

Georg Merling and Hugo Kohler, Elberfeld, Ger. Assignors to Farbenfabriken vorm Friedr Bayer & Co., Elberfeld, Ger. 1,036,876. Process of Producing Erythrene.

Georg Merling and Hugo Kohler, Elberfeld, Ger. Assignors to Farbenfabriken vorm Friedr. Bayer & Co., Elberfeld, Ger. 1,036,876. Process of Producing Erythrene.

Carl Mollenhoff, Leverkusen, Near Cologne, Ger. Assignor to Farbenfabriken, vorm Friedr. Bayer & Co., Elberfeld, Ger. 1,036,880. Production of Alizarin.

Henry Tideman, Menominee, Mich. 1,036,933. Transmitter for the Deaf.

J. D. Knowlton, Westbrook, Me. 1,037,023. Druggist's Device for Dividing Powers.

E. H. Strickler, New York, N. Y. Assignor to General Chemical Co. 1,037,078. Effervescent Compound.

W. R. Archibald, Selma, Ala. 1,037,102. Thermometer-Case.

John Wemer, New York, N. Y. 13,462. Codin Solution.

J. C. Carbson and O. G. Saxton, Easton, Colo. 1,037,864. Surgical Needle Holder.

Wilhelm Ruppel, Hocst on the Main, Ger. Assignor to Farbwerke Vorm. Meister Lucious & Bruning, Hochst on the Main, Ger. 1,037,997. Process of producing a Remedy for Tuberculosis.

A. C. Snell, Rochester, N. Y. Assignor to Electro Surgical Instrument Co., Rochester, N. Y. 1,038,011. Tool Holder.

Heinrich Thron, Frankfort on the Main, Ger. Assignor to The Firm of Vereinigte Chinifabriken Zimmer & Co., G. M. B. H. Frankfort on the Main, Ger. 1,038,027. Manufacture of Hydroquinin.

J. F. Kruse, Denver, Colo. 1,038,394. Portable Vapor Bath Cabinet.

Gustave Fernekes, Pittsburgh, Pa. Assignor to J. A. Snee, Elizabeth, Pa. 1,038,546. Apparatus for Producing Formal Dehyde.

